# Serum Concentrations of Chemokines CCL20, CXCL8 and CXCL10 in Relapsing-Remitting Multiple Sclerosis and Their Association with Presence of Antibodies against Epstein–Barr Virus

**DOI:** 10.3390/ijms25158064

**Published:** 2024-07-24

**Authors:** Jelena Košćak Lukač, Koraljka Bačić Baronica, Alan Šućur, Josip Sremec, Sanja Tomasović, Robert Baronica, Tomislav Kelava, Danka Grčević, Nataša Kovačić

**Affiliations:** 1 Department of Neurology, Clinical Hospital “Sveti Duh”, Sveti Duh 64, 10 000 Zagreb, Croatia; koraljka7@yahoo.com (K.B.B.); joza_sremec@yahoo.com (J.S.); stomasovic98@gmail.com (S.T.); 2 Neurology Clinic, Faculty of Medicine, Josip Juraj Strossmayer University of Osijek, Josipa Huttlera 4, 31 000 Osijek, Croatia; 3 Laboratory for Molecular Immunology, Croatian Institute for Brain Research, Šalata 12, 10 000 Zagreb, Croatia; alan.sucur@mef.hr (A.Š.); tomislav.kelava@mef.hr (T.K.); danka.grcevic@mef.hr (D.G.); natasa.kovacic@mef.hr (N.K.); 4 Department of Physiology and Immunology, University of Zagreb School of Medicine, Šalata 3, 10 000 Zagreb, Croatia; 5 Department of Anesthesiology, Reanimatology, Intensive Medicine and Pain Therapy, University Hospital Centre Zagreb, Kišpatićeva 12, 10 000 Zagreb, Croatia; rbaronica@gmail.com; 6 Department of Anatomy, University of Zagreb School of Medicine, Šalata 11, 10 000 Zagreb, Croatia

**Keywords:** multiple sclerosis, EBV, CCL20, CXCL8, CXCL10

## Abstract

Epstein–Barr virus (EBV) infection and various chemokines, including CCL20, CXCL8 and CXCL10 are considered to participate in the pathogenesis of multiple sclerosis (MS), and several studies point to a direct regulatory effect of EBV on the expression of these chemokines. In our study we hypothesized that serum concentrations of CCL20, CXCL8 and CXCL0 are induced in patients with relapsing-remitting MS (RRMS) in comparison to healthy individuals, and that they are associated with EBV infection. Serum concentrations of CXCL8 and CXCL10 were lower in RRMS patients in relapse in comparison to healthy controls. Although potential effects of corticosteroid therapy introduced in a subgroup of RRMS patients prior to sampling were excluded by subgroup comparison, this possibility has to be considered while interpreting the results. We found an inverse association between serum concentrations of CXCL8 and anti-Epstein–Barr Virus Nuclear Antigen (EBNA) IgG and decreased expression of CXCL8 in peripheral blood mononuclear cells (PBMC) in relapse compared to remission. Lower serum concentrations of CXCL8 and CXCL10 in RRMS patients and decreased peripheral production of CXCL8 in relapse may indicate compensatory anti-inflammatory counter-regulation in MS.

## 1. Introduction

Multiple sclerosis (MS) is an immune-mediated inflammatory and demyelinating disease of the central nervous system (CNS). In its initial phase, the disease is characterized by the influx of peripheral autoreactive lymphocytes to CNS across the blood–brain barrier (BBB), which leads to inflammation and tissue damage [1,2]. 

The etiology of MS includes genetic and environmental factors. Among environmental factors, Epstein–Barr virus (EBV) infection, sunlight exposure, levels of vitamin D, and smoking are often considered to be associated with pathogenesis of MS [3]. EBV is a member of the human herpesvirus family which establishes a latent life-long infection, persisting in resting memory B cells [4]. Meta-analyses confirmed higher frequency of EBV-seropositivity among patients with MS than in healthy controls. However, prevalence of EBV seropositivity is also high among the general population [5,6], which implies the participation of other risk factors in disease manifestation [3]. Despite various proposed mechanisms, determining the exact role of EBV in MS development still remains challenging. Furthermore, at the moment it is not clear whether lytic or specific latent phases of EBV infection plays a role in MS pathogenesis [7]. 

Many chemokines are implicated in the pathogenesis of MS [8,9,10]; however, only CCL20 (C-C motif ligand 20; MIP 3-α, macrophage inflammatory protein-3; LARC, liver and activation regulated chemokine), CXCL8 (IL(interleukin)-8, C-X-C motif ligand 8) and CXCL10 (C-X-C motif ligand 10; IP-10, interferon -induced protein 10 kDa) expressions are induced by EBV in conditions other than MS [11,12,13,14,15,16].

Despite the lack of evidence of direct effects of EBV infection on the expression of these chemokines in MS, their alterations have been previously associated with MS pathogenesis [8,10]. CCL20 is produced by various cells including neutrophils, natural killer (NK) cells, B lymphocytes, T helper 17 (Th17) lymphocytes, macrophages, and dendritic cells [17]. CCL20, as a sole ligand of CCR6 (C-C motif chemokine receptor 6), attracts lymphocytes [18,19], and the CCR6–CCL20 axis is important for the initiation of experimental autoimmune encephalomyelitis (EAE), by promoting the infiltration of IL-17 cells to CNS via the choroid plexus [20,21]. Ambrossini et al. demonstrated that astrocytes, as an integrative part of the blood–brain barrier, produce CCL20 and recruit leukocytes to CNS in relapses of EAE [22]. In addition, immunization against CCL20 ameliorates EAE [20]. 

CXCL8 is produced by numerous cells, including macrophages, lymphocytes T, monocytes, endothelial cells and fibroblasts [23]. CXCL8 plays an important role in activation and migration of neutrophils during inflammation [23,24]. Increased expression of CXCL8 was detected in astrocytes situated in the marginal areas of active MS lesions, while on oligodendrocytes from MS patients upregulated expression of CXCL8 receptors CXCR(C-X-C motif chemokine receptor)1 and CXCR2 was observed [25]. In addition, CXCR2 is expressed by the endothelium of brain vessels, and its activation by CXCL8 induces dysregulation of the BBB [26]. Moreover, it has been demonstrated that the blocking of CXCR2 in EAE prevents breakdown of BBB and infiltration of CNS by leukocytes [27]. CXCL10 is a chemokine expressed by leukocytes, including neutrophils and monocytes, epithelial and endothelial cells. Secretion of CXCL10 is stimulated by interferon (INF)-γ [28]. CXCL10 is a chemoattractant for activated Th1 lymphocytes, monocytes and NK cells. It also promotes endothelial adhesion [29,30]. CXCL10 and its receptor CXCR3 play an important role in the accumulation of T cells in the CNS of MS patients [31]. Hence, application of anti-CXCL10 decreases mononuclear infiltration of CNS and diminishes severity of EAE [32]. Furthermore, incubation with serum-derived EBV (+) exosomes of MS patients induces secretion of CXCL10 by monocyte-derived macrophages [33]. 

So far, concentrations of CCL20, CXCL8 and CXCL10 in serum and in the cerebrospinal fluid (CSF) of MS patients have been analyzed sporadically, using a diverse methodology and comparison groups, so currently available data are not conclusive [34,35]. Furthermore, a very small number of studies have dealt with the potential association between EBV and chemokines CXCL10 and CXCL8 in MS so far [33,36], and per our knowledge, none of the studies investigated correlation between EBV and CCL20. In our study we hypothesize that peripheral concentrations of CCL20, CXCL8, and CXCL10 are increased in MS patients in relapse in comparison to healthy controls. We also hypothesize that EBV infection as a trigger for MS will increase expression of these chemokines. Therefore, we compared serum concentrations of CCL20, CXCL8, and CXCL10 in RRMS patients in relapse with their concentrations in healthy controls and explored the association of chemokines with clinical characteristics of the disease, and concentrations of anti-EBV antibodies in patients with relapsing-remitting MS (RRMS).

## 2. Results

### 2.1. Concentrations of CXCL10, and CXCL8 Are Decreased in MS Patients

**Figure 1:**
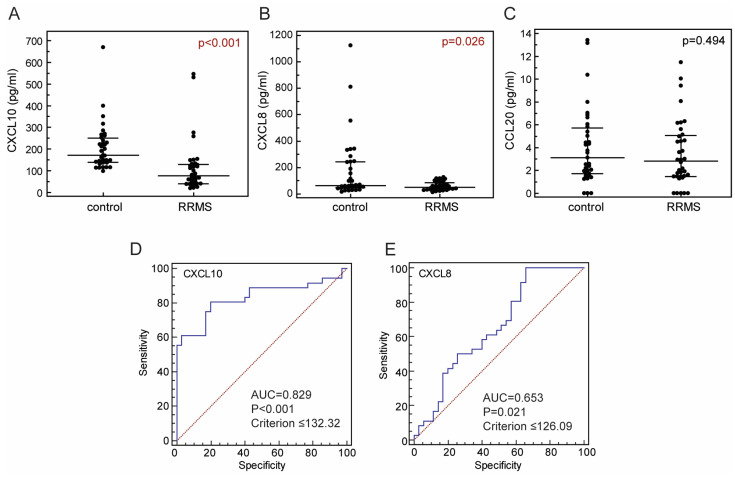
Concentrations of CXCL10 (**A**), CXCL8 (**B**) and CCL20 (**C**) in the sera of control subjects (N = 35) and patients with relapsing-remitting multiple sclerosis in relapse (RRMS; N = 36). Ability of concentrations of CXCL10 (**D**) and CXCL8 (**E**) in the sera to discriminate between RRMS patients in relapse and control subjects. Concentrations were determined using a cytometric bead-based array (LEGENDplex™). Markers represent individual values, horizontal lines and bars are median and IQR (**A**–**C**). Red lines represent reference lines, blue lines represent ROC curves (**D**,**E**). Statistical significance (*p* values), area under curve (AUC), sensitivity, specificity and “cut off” point (criterion) is marked on plots; Mann–Whitney test (**A**–**C**) or ROC curve analysis (**D**,**E**). Significant statistical difference is written in red.

We first compared concentrations of selected chemokines in the sera of patients in MS relapse with their concentrations in the sera of control subjects, and detected significantly lower concentrations of CXCL10 and CXCL8 in RRMS patients (CXCL10, 76.49 [40.17–128.90] pg/mL; CXCL8, 50.16 [34.25–86.61] pg/mL) in comparison to controls (CXCL10, 171.40 [138.31–249.79] pg/mL; CXCL8, 64.02 [47.05–242.34] pg/mL; *p* < 0.001; *p* = 0.026, respectively, Mann–Whitney test, Figure 1A,B). Serum concentration of CCL20 did not differ between patients (2.84 [1.48–5.06] pg/mL) and control subjects (3.13 [1.71–5.72] pg/mL, *p* = 0.494; Mann–Whitney test, Figure 1C). Additionally, concentrations of CXCL10 and CXCL8 were able to discriminate between RRMS patients in relapse and healthy controls (Figure 1D,E).

Within the group of RRMS patients in relapse, samples were, in 11 patients, collected before the initiation of corticosteroid therapy, while in 25 patients, samples were collected within 24 h after the initiation of corticosteroid therapy. We suspected that early response to corticosteroid therapy might have influenced our results, so we compared serum concentrations of chemokines in groups of patients who did not receive corticosteroids with samples from patients taken after the initiation of therapy and found no significant difference in any of the chemokines regarding the therapy (Table 1). 

Furthermore, in order to exclude the possible influence of corticosteroid therapy, we compared only samples from RRMS patients in relapse without therapy and samples from healthy control subjects, and the pattern of differences in chemokine concentrations remained the same (CXCL8 36.30 [30.07–58.42] in RRMS vs. 64.02 [47.05–242.34] in controls, *p* = 0.019; CLX10 116.79 [47.58–126.54] in RRMS vs. 171.40 [138.31–249.79] in controls, *p* = 0.002, Mann–Whitney test).

The next step was to assess the potential association of chemokine concentrations with patients’ characteristics and disease activity. All three analyzed chemokines were not associated with patients’ age, disease duration, annual relapse rate, EDSS score, number of experienced relapses, or number of relapses in the first two years of the disease. Only CXCL10 concentration was positively associated with the length of the interval between the two latest relapses (ρ = 0.449, *p* = 0.015). Furthermore, concentrations of CXCL8, CXCL10 and CCL20 in sera did not significantly differ among subgroups of MS patients divided by presence or localization of demyelinating lesions (supratentorial, infratentorial and cervical), sex, or history of optic neuritis. 

### 2.2. CXCL10 and CXCL8 Decrease during Relapse

Despite significantly lower peripheral concentrations of CXCL8 and CXCL10 in RRMS patients in relapse than in control subjects, their association with disease characteristics or activity was not established in the initial study population, which may be ascribed to patients’ heterogeneity. Therefore, to clearly assess the potential association of selected chemokines with the activity of RRMS, we recruited a cohort of patients in which we harvested a blood sample during a relapse, and a second sample after the same patient has entered a remission. In these paired samples (from the same patient) we measured the serum concentration and expression of genes for selected chemokines in PBMC, which allowed us to assess the peripheral production of analyzed chemokines. 

**Figure 2:**
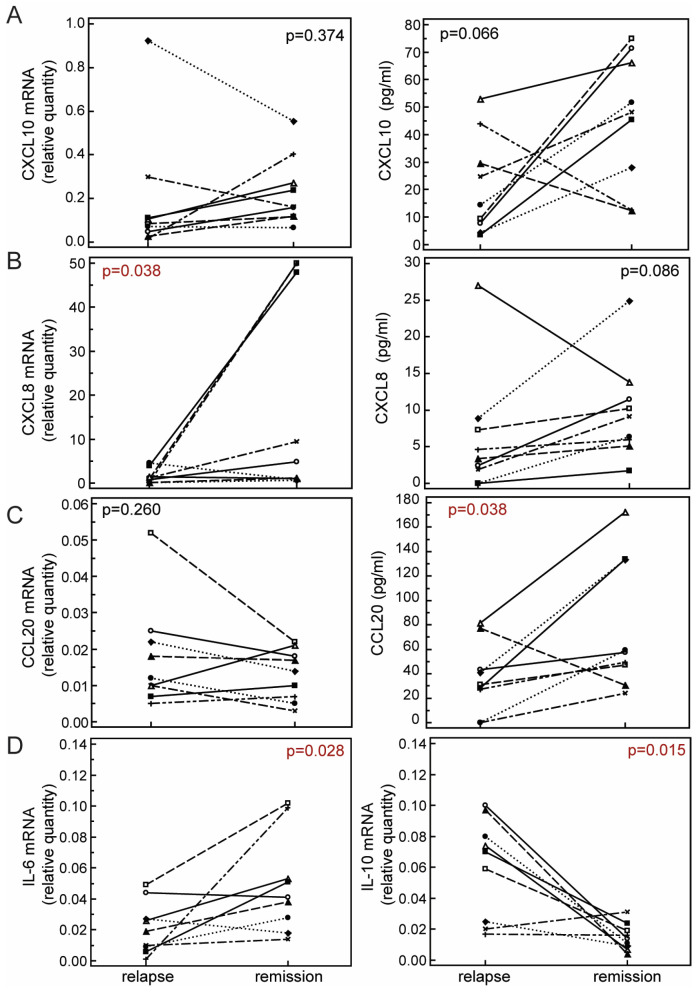
Gene expression in peripheral blood mononuclear cells (left panels) and serum concentration (right panels) of CCL20 (**A**), CXCL8 (**B**), and CXCL10 (**C**), and gene expression of IL-6 (lowest left panel) and IL-10 (lowest right panel) (**D**) during relapse (N = 9) and remission (N = 9) in paired samples of patients with relapsing-remitting multiple sclerosis (RRMS). Markers represent individual patients’ values. Analysis was performed using Wilcoxon test. Statistical significance is marked on the plots (*p* values); significant statistical difference is written in red.

Gene expression of CXCL8 and CXCL10 was, in the majority of assessed patients, higher in remission than in relapse (8/9 for CXCL8, and 7/9 for CXCL10). However, the difference was statistically significant only for the gene expression of CXCL8 (Figure 2A,B). Serum concentration of CCL20 showed a similar pattern as other chemokines, and was significantly lower in relapse than in remission, but its gene expression was similar in both phases (Figure 2C). 

As an increase in inflammatory activity is a hallmark of MS relapse, our findings were somehow unexpected. To validate the findings, we also assessed gene expression of two established cytokine markers of inflammatory activity; IL-6, a pleiotropic proinflammatory cytokine [37]; and IL-10, an anti-inflammatory cytokine [38]. Expression of IL-6 was clearly reduced in relapse (0.02 [0.01–0.03]) in comparison to remission (0.04 [0.03–0.09], *p* = 0.028, Wilcoxon test), while expression of IL-10 was increased in relapse (0.07 [0.02–0.09]) in comparison to remission (0.01 [0.01–0.02], *p* = 0.015, Wilcoxon test, Figure 2D). Taken together, the results indicate reduced peripheral inflammatory activity in RRMS patients during relapse, which might represent a counter-regulatory mechanism, as an attempt to limit the inflammatory activity in the CNS. 

### 2.3. Frequency of Anti-EBV Antibodies Is Similar in Controls and RRMS Patients but Concentration of Anti-EBNA Is in RRMS Patients Inversely Associated with Concentration of CXCL8

Anti-Epstein–Barr Virus Nuclear Antigen (EBNA) and anti-Viral Capsid Antigen (VCA) IgG antibodies were detected in the majority of subjects in similar frequencies (Table 2). Anti-VCA IgM and anti-Early Antigen (EA) IgG were rarely detected and more frequent amongst control subjects.

**Figure 3:**
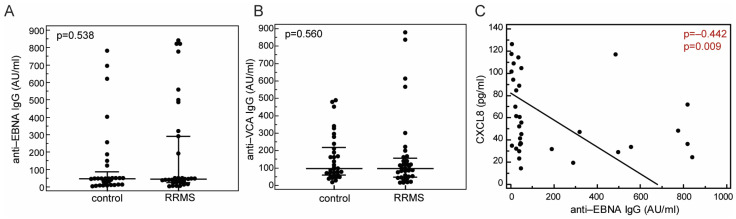
Concentrations of anti-EBNA IgG (**A**) and anti-VCA IgG (**B**) in healthy control subjects ((**A**), N = 32, (**B**), N = 33) and patients undergoing a relapse of relapsing-remitting multiple sclerosis (RRMS; (**A**), N = 34, (**B**), N = 35), and the association (**C**) of concentrations of CXCL8 and anti-EBNA IgG antibodies in the sera collected from group of patients with relapsing-remitting multiple sclerosis in relapse (RRMS, N = 34). Markers represent individual values, horizontal lines and bars are median and IQR, statistical significance (*p*) and Spearman’s rank correlation coefficient (ρ) are marked on plots. Significant statistical difference is written in red.

Concentrations of anti-EBNA IgG and anti-VCA IgG did not differ between RRMS patients (anti-EBNA IgG 43.60 [25.52–290.28] AU/mL; anti-VCA IgG, 97.02 [47.67–156.53] AU/mL) and controls (anti-EBNA IgG, 46.53 [11.48–86.17] AU/mL; anti-VCA IgG, 95.68 [58.90–218.01] AU/mL, *p* = 0.538; *p* = 0.560, respectively, Mann–Whitney test, Figure 3A,B). Furthermore, concentrations of anti-EBV antibodies were not associated with disease characteristics. In addition, we separately assessed the associations of anti-EBV antibodies with chemokine concentrations within the RRMS group and the healthy control group of the cross-sectional cohort. In the RRMS group, serum concentration of anti-EBNA IgG was inversely associated with the serum concentration of CXCL8 (ρ = −0.442, *p* = 0.009, Figure 3C), while this association was absent in the group of healthy control individuals (ρ = 0.202, *p* = 0.267), which might suggest the specific effects of EBV infection on immune cell trafficking and CXCL8 secretion in RRMS. Concentration of anti-EBNA IgG was not associated with concentrations of CXCL10 (ρ = −0.134, *p* = 0.451) and CCL20 (ρ = 0.004, *p* = 0.982) in the RRMS group, nor in healthy control subjects (CXCL10, ρ = −0.047, *p* = 0.788; CCL20, ρ = 0.162, *p* = 0.353). Concentration of anti-VCA IgG was not associated with any of the selected chemokines in RRMS patients (CXCL8, ρ = −0.169, *p* = 0.330; CXCL10, ρ = 0.152, *p* = 0.385; CCL20, ρ = −0.004, *p* = 0.981).

## 3. Discussion

According to our study, serum concentrations of CXCL8 and CXCL10 decrease in RRMS patients during relapse, while concentrations of CCL20 are similar to those of healthy subjects. Analysis of paired samples showed that gene expression of CXCL8 in PBMC decreases in relapse in comparison to remission, pointing to its reduced peripheral production. Serum concentrations of CCL20 are lower in relapse in comparison to remission, but its gene expression is similar in relapse and remission, pointing to other regulatory mechanisms, such as migration of peripheral CCL20-producing cells to CNS. These findings, together with the reduced expression of IL-6 and increased expression of IL-10 in relapse, indicate potential counter-regulatory mechanisms as an attempt to limit the inflammatory activity in the CNS.

Some of the previous studies support our findings reporting decreased concentration of CXCL8 in patients with MS [39,40,41]. Matejčikova et al. reported significantly lower serum levels of CXCL8 in MS patients than in controls, and significantly higher CSF levels of CXCL8 in MS patients in comparison to controls, which was positively associated with Q-albumin, a marker of BBB damage [39]. Other studies contrast our findings, reporting significantly higher serum levels of CXCL8 among MS patients in comparison to controls [36,42,43]. Among them, only a study reported by Bartosik-Psujek et al. enrolled RRMS patients during relapse, so contradictory results may result from different inclusion criteria. A few earlier studies reported increased or similar serum concentrations of CXCL10 in RRMS patients during relapse and controls [44,45]. While reporting no difference in concentrations of CXCL10 in sera of RRMS patients and controls, Sinder and co-workers observed decreased expression of its receptor, CXCR3, in peripheral blood T-cells from RRMS patients, and an increased concentration of CXCL10 in CSF during relapse [46]. While some of the previous studies support our findings regarding a lack of differences between serum concentrations of CCL20 in RRMS patients during relapse and controls [47,48], others reported significantly higher serum concentrations of CCL20 in RRMS patients in relapse in comparison to controls [49,50]. These findings together point to alterations of chemokine regulatory networks in patients with MS which still need to be precisely defined. Furthermore, our results showed that concentrations of CXCL10 and CXCL8 were able to discriminate between RRMS patients in relapse and healthy controls, which may point to a possible diagnostic value of these chemokines. Concentrations of those chemokines followed the same pattern in paired samples from patients in relapse and remission, so increases in CXCL8 and CXCL10 concentration above the criterion might be used as a recovery marker; however, further studies on larger numbers of participants are required to confirm that notion.

Infection with EBV has, for a long time, been considered as an important contributor for the development of MS, and a recent assessment by Bjornevik et al. reports a 32-fold increase in the risk for MS development [51,52]. In our study, anti-EBV antibodies were detected in all MS patients enrolled in our study. In the majority of patients, we found the presence of anti-VCA IgG and anti-EBNA IgG, which indicates past infection [53]. Our results are in accordance with previous epidemiological study on Croatian population, which reported the presence of anti-VCA IgG in 100% of MS patients and 95.9% of the general adult population. Moreover, the latter study reported the prevalence of anti-VCA IgM seropositivity among the general population aged 20–29 years as 10.6% [54]. This could explain our results of anti-VCA IgM prevalence among controls, taking into consideration the age of the controls, among which were also participants younger than 30 years of age. 

Unlike our findings, previous meta-analyses reveal an increased frequency of anti-EBNA IgG antibodies and anti-VCA IgG antibodies in MS patients in comparison to controls. According to one of the meta-analyses, median seropositivity for anti-EBNA IgG antibodies was 98% among MS patients and 88% among controls. Median seropositivity for anti-VCA IgG antibodies was also higher in MS patients (99%) than in controls (92%) [5]. Another meta-analysis reported an overall EBV-seroprevalence of 92.9% among MS patients and 85.6% among control adult subjects [6]. In comparison to these meta-analyses [5,6], it seems that the overall EBV-seroprevalence in Croatia is very high. Previous studies also reported higher [36,55,56,57] or similar [58] serum concentrations of anti-EBNA-1 IgG in MS patients in comparison to controls. However, previous studies that assessed serum levels of anti-VCA IgG in MS patients are concordant with our findings [55,59], but also report higher levels of anti-VCA IgG in MS patients in comparison to controls [60]. The lack of differences in our study could be explained by very high overall EBV-seroprevalence among the Croatian population in comparison to mentioned meta-analyses, as well as by a relatively small sample size.

One of the aims of our study was to assess the association between EBV infection and three selected, EBV-regulated chemokines, CCL20, CXCL8 and CXCL10 in MS. Our study revealed only modest inverse association of concentrations of CXCL8 and anti-EBNA IgG in RRMS patients during relapse, but not in the control individuals. However, this should be taken with caution, since no difference in concentrations of antibodies were observed, as well as because the majority of MS patients and control participants were seropositive. 

Some results of our study should be interpreted while taking into account its limitations. The main limitation is the small number of participants, especially for the analysis of CCL20 and anti-EBV antibodies, so further studies are required to validate the results on a larger number of participants, including populations with lower prevalence of EBV seropositivity than the Croatian population. In addition, despite the lack of significant differences in chemokine concentration between untreated patients and patients treated with corticosteroids, due to the small number of samples taken prior to introduction of corticosteroid therapy, the effect of corticosteroids cannot be fully excluded. However, it is important to note that these samples were taken very early (within 24 h after the initiation of corticosteroid therapy), so due to the delayed onset of corticosteroid therapy these effects are unlikely [61]. Paired samples were collected during relapse and remission. Since DMT alters inflammatory cytokine expression [62,63,64,65,66] we preferred the inclusion of patients who are DMT-naïve. Current treatment practices and insurance policies in Croatia support the introduction of DMT as early as possible after RRMS diagnosis, which made it difficult to collect samples from DMT-naïve patients, especially in the cohort of paired samples. Another limitation of the study is the absence of CSF samples due to the lack of indication for lumbar puncture. In addition, analysis of CSF concentration of CXCL10, CXCL8 and CCL20 could provide useful data on relation and dynamics between periphery and CNS in pathogenesis of MS. As lumbar puncture is rarely indicated, precise establishment of chemokine markers such as CXCL8 may help to discriminate between patients in relapse and in remission.

In summary, we found lower serum levels of CXCL8 and CXCL10 in RRMS patients in comparison to controls, inverse association of concentration of CXCL8 with anti-EBNA IgG, and decreased peripheral production of CXCL8 in relapse, which may be explained by compensatory anti-inflammatory counter-regulation mechanisms in MS, and further studies are required to precisely define these mechanisms.

## 4. Materials and Methods

### 4.1. Patients

**Figure 4:**
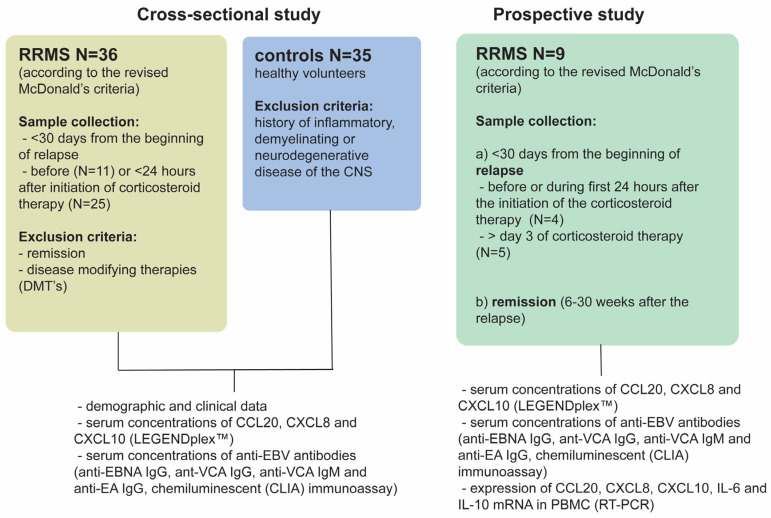
Schematics of the study design. The cross-sectional part of the study included 36 patients with RRMS admitted to Clinical Hospital ‘Sveti Duh’, within 30 days from last relapse. The diagnosis of RRMS was established according to the revised McDonald’s criteria [67]. Patients in remission and those who were taking disease-modifying therapies (DMT’s) before or at the time of sample collection were excluded from the study. Samples were collected either before (N = 11) or within 24 h after initiation of corticosteroid therapy (N = 25). Healthy volunteers (N = 35), matched by age and gender to MS patients, without history of inflammatory, demyelinating or neurodegenerative disease of CNS were also included in the study. The prospective part of the study included an additional 9 patients whose blood samples were collected during relapse and after they achieved remission (6–30 weeks after the relapse). In both cross-sectional and prospective groups’ samples, sera were used to determine the concentrations of chemokines and anti-EBV antibodies. In the prospective group gene expression of chemokines was additionally assessed in peripheral blood mononuclear cells (PBMC). Four samples from the prospective group in relapse were collected before or during the first 24 h after the initiation of the corticosteroid therapy, while five samples were collected on or after the third day of 5-day pulse corticosteroid therapy. Expression levels of assessed chemokines and cytokines were not significantly different between these two groups of patients in relapse.

The study consisted of 2 parts, cross-sectional and prospective, schematically presented in Figure 4.

Participants were enrolled in the study after signing informed consent, following ethical approval obtained from the Ethics Committees of Clinical Hospital ”Sveti Duh” (approval number 01-6953/13 and 012-4445; approval date 1 June 2017. and 3 September 2020). The study was conducted in accordance with the Declaration of Helsinki.

The following data were collected from patients or recorded from their medical documentation: age and sex, duration of the disease, Expanded Disability Status Scale (EDSS) score before the onset of relapse, number of experienced relapses, number of relapses per year, number of relapses in the first two years of the disease, time elapsed from the previous relapse, initial and current symptoms (categorized as sensory, motor and brainstem symptoms), presence of optic neuritis at current relapse, presence of oligoclonal bands in CSF and MRI findings, localization of the lesions (supratentorial, infratentorial, cervical and/or thoracic) and presence of gadolinium-enhancing lesions. All MRI scans were acquired using a 1.5 T MR scanner (Koninklijke Philips N.V., Eindhoven, The Netherlands). Characteristics of patients and controls are presented in Table 3.

### 4.2. Sample Collection and Measurement of Chemokines 

Venous blood (4 mL) was collected into vials with a clot activator, centrifuged for 10 min/2000 rpm, and after 30 min, sera were collected and stored at −20 °C. 

Concentrations of CCL20, CXCL8 and CXCL10 were assessed in undiluted sera by flow-cytometric bead-based array LEGENDplex™ Human Mix and Match Proinflammatory Chemokine Panel 1 (Biolegend, San Diego, CA, USA) using CCL20 capture bead B5, CXCL10 capture bead A5, and CXCL8 capture bead A4), as described previously [68]. Briefly, sera were incubated with beads coated with a capture antibody specific for chemokine. Samples were then washed and incubated with the secondary antibody conjugated with biotin, and detected by streptavidin-PE. Chemokine concentration was determined by the intensity of the PE fluorescence. Fluorescence intensity was measured by Attune flow cytometer (Applied Biosystems, Thermo Fisher Scientific, Waltham, MA, USA), and the concentration of each chemokine was determined using LEGENDplex™ data analysis software v8.0 (BioLegend, https://legendplex.qognit.com, accessed on 14 September 2019 and 14 October 2022), based on a standard curve generated by data acquired from chemokine standards provided within the kit. 

### 4.3. Measurement of Antibodies to EBV

Anti-EBV antibodies (anti-EBNA IgG, anti-VCA IgG, anti-VCA IgM and anti-EA IgG) were measured by the chemiluminescent immunoassay (CLIA) method (Maglumi, Snibe Co., Ltd., Shenzhen, China), according to manufacturer’s recommendations. The light signal of the chemiluminescent reaction was measured by photomultiplier as relative light units (RLU) proportional to EBV antibody concentration in sample. EBV antibody concentration was determined using calibration curve and the results were expressed as arbitrary units (AU)/mL. If the recorded value was 50 AU/mL (upper limit value), serum sample was diluted with PBS (1:21) and the analysis was repeated as described previously. 

### 4.4. RNA Extraction and Real-Time PCR

Peripheral blood mononuclear cells (PBMC) were separated using Histopaque (Sigma-Aldrich, St. Louis, MO, USA). Total RNA was extracted using the TRIzol reagent (Applied Biosystems, Thermo Fisher Scientific, Waltham, MA, USA), [69] reversely transcribed (1 μg) to cDNA and amplified (20 ng/well) by quantitative (q) PCR using an AB7500 (Applied Biosystems) instrument, and commercially available TaqMan Assays (Hs00174103_m1 Human CXCL8, Hs00355476_m1 Human CCL20, Hs00171042_m1 Human CXCL10, Hs00985639_m1 Human IL-6, Hs00961622_m1 Human IL-10, Applied Biosystems), as described previously [70,71]. The relative quantities of unknown samples were calculated by using the standard curve created by 6 serial dilutions of the calibrator sample (control PBMC) normalized to GAPDH as the endogenous control. 

### 4.5. Data Analysis

Clinical data were presented as median and interquartile range (IQR) and compared using the non-parametric Mann–Whitney or Fisher’s exact test. Associations between variables were analyzed by Spearman’s rank correlation. Data from paired samples collected during relapse and remission were compared by Wilcoxon test. All tests were performed using MedCalc software, version 22.023 (MedCalc Software Ltd., Ostend, Belgium), and significance level was set at 0.05. 

## Figures and Tables

**Table 1:** Concentrations of CXCL10, CXCL8 and CCL20 in sera from RRMS patients in relapse who did not receive corticosteroids before sampling, and patients who received corticosteroids <24 h prior to sampling.

Chemokine	Concentration without Therapy (N = 11) *	Concentration with Therapy (N = 25) *	*p* **
CXCL10	116.79 [47.58–126.54]	67.44 [40.26–131.33]	0.548
CXCL8	36.30 [30.07–58.42]	58.33 [36.11–102.21]	0.110
CCL20	2.03 [1.66–4.15]	3.00 [1.47–5.75]	0.659

Data are presented as median and interquartile range, * pg/mL, ** Mann–Whitney test.

**Table 2:** Frequencies of anti-EBV antibodies in control subjects and patients with relapsing-remitting multiple sclerosis (RRMS) in relapse.

	Control (N = 35)	RRMS (N = 36)	*p* *
Anti-EBNA IgG (n, %)	32 (91.42%)	34 (94.44%)	0.674
Anti-VCA IgG (n, %)	33 (94.29%)	36 (100%)	0.239
Anti-VCA IgM (n, %)	5 (14.29%)	0 (0%)	0.025
Anti-EA IgG (n, %)	4 (6.15%)	1 (2.85%)	0.199

* Fisher’s exact test.

**Table 3:** Patients’ demographic and clinical data.

	MS (N = 36)	Control (N = 35)
Sex (female/male)	26/10	26/9
Age (female/male)	37.0 [29.5–47.5] (40.5 [33.0–49.0]/28.5 [26.0–37.0])	38.0 [31.0–53.0] (41.5 [31–53]/35.0 [30.75–47.75])
EDSS score (female/male)	1.75 [0–2.75] (1.75 [0–3.0]/1.75 [0–2.0])	n/a
Duration of the disease (years, female/male)	5.5 [1.0–8.5] (6 [1.0–11.0]/3.5 [0–6.0])	n/a
Total number of experienced relapses (female/male)	2.0 [1.0–3.0] (3.0 [2.0–3.0]/2.5 [2.0–4.0])	n/a
Annualized relapse rate (female/male) *	0.47 [0.318–1.042] (0.445 [0.280–0.750]/1.17 [0.425–2.112])	n/a
Number of relapses in first two years of the disease (female/male) ** Time elapsed from previous relapse (female/male) *	1.0 [1.0–2.0] (1.0 [1.0–2.0]/2 [1.0–3.0]) 18.0 [10.75–72.0] (22.5 [16.0–84.0]/14.0 [5.25–40.0])	n/a n/a

Data are presented as median [IQR], * N = 29 (22/7), ** N = 26 (20/6); n/a, not applicable.

## Data Availability

Data will be available upon request.
